# Retrospective Evaluation of the Topical Use of 1% Amitriptyline in Patients with Burning Mouth Syndrome

**DOI:** 10.3390/dj14060317

**Published:** 2026-05-22

**Authors:** Carmen Steffani Perez-Parrella, Juan Antonio Ruiz Roca, Eduardo Pons-Fuster, Pia López-Jornet

**Affiliations:** 1 Department of Dermatology, Stomatology, Radiology and Physical Medicine, Faculty of Medicine, University of Murcia, and Biomedical Research Institute of Murcia (IMIB), Ctra. Madrid-Cartagena, s/n, El Palmar, 30120 Murcia, Spain; carmensteffani.perezp@um.es (C.S.P.-P.); jaruizroca@um.es (J.A.R.R.); 2 Department of Human Anatomy, University of Murcia El Palmar, 30120 Murcia, Spain; eduardo.p.f@um.es

**Keywords:** BMS, topical, treatment, amitriptyline

## Abstract

**Objective**: The aim of this study was to explore the clinical response and tolerability of topical 1% amitriptyline in patients with burning mouth syndrome in a real-world clinical setting. **Methodology**: A retrospective observational study was conducted through a review of the clinical histories of patients diagnosed with burning mouth syndrome, treated at the Dental Clinic of the Morales Meseguer Hospital (Murcia, Spain). All the patients were treated with topical amitriptyline for a period of four weeks. The following parameters were evaluated at the start of the treatment and at the end of the four weeks: pain or mouth burning, through the visual analog scale (VAS); anxiety and depression, with the Hospital Anxiety and Depression (HAD) scale; sleepiness, with the Epworth Sleepiness Scale (ESS); sensation of dry mouth (VAS) and basal sialometry for the objective measurement of salivary flow. **Results**: Of the 32 patients that were initially included, 27 were ultimately analyzed. After 4 weeks of treatment with 1% topical amitriptyline, a significant improvement was observed in mouth pain or burning, measured with the VAS, with a decrease in the median of 7.5 (IQR 6–9) to 5 (IQR 5–7) (*p* < 0.001). Likewise, significant improvements were recorded in the anxiety (HAD-A) and depression (HAD-D) scores, with significant reductions after the treatment (*p* = 0.019 and *p* = 0.009, respectively). No statistically significant differences were observed in the subjective sensation of dry mouth (VAS) (*p* = 0.054) or in the total production of saliva (*p* = 0.477). **Conclusions**: Treatment with 1% topical amitriptyline for four weeks was associated with a reduction in pain and emotional distress in patients with burning mouth syndrome, with very few reported adverse effects. As an exploratory retrospective study with a limited sample size reflecting real-world clinical practice, these findings suggest that 1% topical amitriptyline may represent a useful therapeutic option in the management of burning mouth syndrome. However, the results should be interpreted with caution, and further prospective controlled studies are needed to confirm these findings.

## 1. Introduction

Burning mouth syndrome (BMS) is a chronic orofacial pain disorder. According to the International Classification of Orofacial Pain, it is defined as an intraoral sensation of burning or dysesthesia that is present daily for more than two hours a day and for a period longer than three months, without evident causal lesions during clinical exploration or complementary tests [1]. It is more frequent in women, and its prevalence is variable according to the population studied [2].

BMS is still a therapeutic challenge, mainly due to the absence of standardized treatments and the variability in the clinical response [3,4]. In odontological practice, many alternatives have been used with different results, both pharmacological (benzodiazepines, gabapentin, clonazepam, tricyclic antidepressants, capsaicin, antioxidants) and non-pharmacological (photobiomodulation, cognitive-behavioral therapy, acupuncture, among others). In the last few years, its management has evolved towards a more comprehensive approach, combining antidepressants, anticonvulsants, and neuromodulators. Among the emerging treatments, vortioxetine is notable, useful in moderate or severe cases due to its action against pain, mood, cognition, and sleep, with a good tolerance even among older patients [5,6].

The therapeutic approach tends to follow a step-by-step approach: in mild cases, topical treatments are started, and if the response is insufficient, systemic options are utilized. Topical treatments seek a local analgesic effect to reduce adverse effects. Among them, clonazepam has been shown to have some efficacy, but its prolonged use can lead to dependency. Capsaicin and topical gabapentin provide some benefits, but their tolerance levels and clinical evidence are variable [3,4,5,6].

Amitriptyline is a tricyclic antidepressant with proven efficacy in neuropathic pain [7]. The initial studies on BMS with amitriptyline, conducted by Fenelon et al. (2017) [8] and Kawasaki et al. (2018) [9], showed a significant reduction in pain with low doses (10–25 mg/day), although with an initial action somewhat slower than clonazepam. Suga et al. [10] confirmed a clinically relevant improvement in 76% of the cases, with a good efficacy even among those older than 75 years old. Posteriorly, Watanabe et al. (2022) compared amitriptyline with aripiprazole in patients ≥80 years old, with the latter demonstrating a higher efficacy and adherence than the former, despite the high incidence of mild adverse effects such as sleepiness, xerostomia, and constipation [11]. More recently, Gonçalves et al. (2024) indicated a rate of response of 74.2% with effective doses of 25–50 mg/day, underlining its efficacy especially in men, although the anticholinergic effects persisted [12]. Due to this limitation, topical administration has gained interest as a safer alternative, as it allows for a local action with minimal systemic absorption. Amitriptyline exerts peripheral effects by blocking sodium and potassium pumps and modulating TRPV1 and NMDA receptors, which support its potential as a local neuromodulator [13,14,15,16,17,18]. Various studies (Lebel et al., 2023, 2024; Hussein & El-Marssafy, 2025) [14,15,16] have shown that 1–2% topical formulations can significantly reduce pain with excellent tolerability, opening a promising therapeutic venue. Given the lack of standardized commercial formulations and the need to consolidate clinical evidence, the aim of the present study was to evaluate the efficacy and tolerability of 1% amitriptyline gel among patients with burning mouth syndrome through a retrospective analysis of clinical histories obtained under conditions of real-world clinical practice.

## 2. Materials and Methods

### 2.1. Study Design and Setting

This is a descriptive, observational retrospective study, conducted with patients who had been diagnosed with burning mouth syndrome (BMS) and treated with a 1% topical amitriptyline that was prepared in an orabase vehicle for four weeks, in conditions of real-world clinical practice, at the Oral Medicine Service of the Dental Clinic at the Morales Meseguer Hospital (Murcia, Spain).

**Figure 1:**
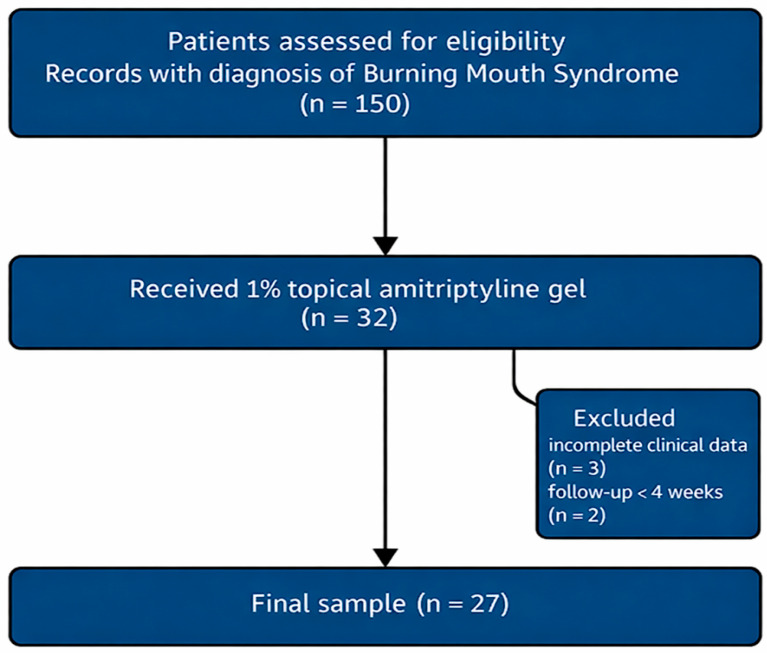
Flowchart of patient selection for inclusion in the retrospective study.

The study protocol was approved by the Ethics Committee of the Morales Meseguer Hospital (code EST: 53/25) and conducted in accordance with the principles of the Declaration of Helsinki. Due to its retrospective design and the use of anonymized clinical data, individual informed consent was not required. The study was retrospectively registered at ClinicalTrials.gov (Identifier: NCT07214181) as an observational retrospective study (Figure 1).

### 2.2. Participants

**Inclusion**

1. Intraoral sensation of burning or persistent dysesthesia for more than 2 h per day and for at least 3 months. BMS diagnosis according to the ICOP 2020 or ICHD-3 diagnostic criteria.
2. Pain with an intensity of ≥4 on a numeric scale (0–10).
3. Continued treatment with 1% topical amitriptyline for at least 4 weeks.

**Exclusion**

The following patients were excluded: those with incomplete clinical data, a follow-up of less than 4 weeks, concomitant use of other topical medications on the oral mucosa, changes in systemic pain medication during the study period, or local or systemic conditions capable of producing symptoms similar to BMS. Secondary causes were ruled out through a detailed medical history, a complete oral examination, and the complementary tests deemed appropriate in routine clinical practice.

### 2.3. Procedure

Clinical records of patients diagnosed with burning mouth syndrome who were treated at the Oral Medicine Service of the Dental Clinic, Morales Meseguer Hospital (Murcia, Spain), were reviewed. Among them, 32 patients had received treatment with a 1% topical amitriptyline gel. After applying the inclusion and exclusion criteria, 27 patients with complete clinical records were finally included in the analysis. The treatment duration was standardized to four weeks for all patients.

According to the documentation of the clinical histories reviewed, the patients received the instructions to apply the 1% amitriptyline gel twice a day directly on the affected area, using a cotton swab or a clean finger. They were given the recommendation to leave the prepared treatment in contact with the area for approximately 2 min, spit the excess without swallowing it, and not rinse the mouth or consume food or drink for at least 30 min after the application. The total duration of the treatment was four weeks, and only the clinical records corresponding to this period were analyzed. The data were collected by a single observer (CS).

### 2.4. Clinical Evaluation and Variables Analyzed

The clinical records of each patient were revised before and during the treatment with the 1% amitriptyline topical gel. The variables included in the analysis were the following:

- **Oral pain or burning:** The mean intensity of pain was recorded with a visual analog scale (VAS, 0–10), in which 0 indicated the absence of pain and 10 the maximum pain imaginable. The measurements were performed at the start, after 15 days, and after 4 weeks of treatment.
- **Anxiety and depression:** These were evaluated with the *Hospital Anxiety and Depression Scale* (HADS-A and HADS-D) [19], applied at the start and after 4 weeks. Scores higher than 10 in each sub-scale were considered indicative of clinical impairment.
- **Daytime sleepiness:** It was determined through the *Epworth Sleepiness Scale* (ESS) [20]. It was applied at the start and at week 4; values ≥ 10 were interpreted as excessive sleepiness.
- **Dry mouth:** This was assessed with a VAS (0–10), in which 0 represents the absence of dryness and 10 the maximum sensation of dry mouth. This was performed in basal conditions and after 4 weeks.
- **Sialometry:** Non-stimulated total salivary flow was determined with the *modified oral Schirmer test* [21,22]. This procedure consists of placing a sterile Schirmer paper strip on the floor of the mouth for 5 min, after which the length of the wet portion is measured and recorded (mm). This was performed in basal conditions and after 4 weeks.
- **Tolerability and acceptance:** All the adverse effects stated by the patients were recorded (mouth dryness, sleepiness, dysgeusia, and others), and the ease of use, taste, or dislike were assessed with a VAS (0–10).
- **Perceived improvement:** At the end of the treatment, patients were asked the direct question: “On a scale of 0 to 10, where 0 means no improvement and 10 means complete improvement, how much do you feel your oral burning or discomfort has improved since the start of treatment?” In addition, the day when the patient stated they perceived the first improvement and the possible appearance of adverse effects was recorded.

### 2.5. Statistical Analysis

The quantitative variables were described with means and standard deviations (SD), or the median and interquartile range (IQR) according to their distribution, as verified with the Shapiro–Wilk test. The comparisons between the pre- and post-treatment measurements were performed with the Wilcoxon signed rank test or Student’s *t* test for related samples, accordingly. **To assess the effect of the treatment, controlling for the clinical variables, mixed-effects linear models were constructed**, considering the longitudinal structure of the data, with repeated measurements obtained at baseline, 15 days, and 4 weeks, considering time (baseline, 15 days, and 4 weeks) **as the main fixed effect and the patients as random effects** to account for within-subject correlation over time. The covariates included in the model were the number of symptomatic locations and the development over time (years). A level of significance of *p* < 0.05 was established. The analysis was performed with the IBM SPSS Statistics program, version 25.0 (IBM Corp, Armonk, NY, USA).

## 3. Results

The final sample was composed of 27 patients who met the inclusion criteria and had complete data for the analysis. The mean age was 63.9 years old (SD = 10.3). Of these, 85% were women (*n* = 23) and 14.8% men (*n* = 4). With respect to the clinical distribution, 59.3% presented more than one symptomatic intraoral location and 40.7% only a single location. The mean time for BMS to develop was 3.53 years (SD = 3.88). As for the clinical developments after four weeks of treatment with amitriptyline, a significant improvement was observed in oral pain or burning, as measured through a VAS scale. The median score decreased from 7.5 (IQR 6–9) to 5 (IQR 5–7) (*p* < 0.001). Likewise, significant improvements were observed in the anxiety and depression scores (HAD-A and HAD-D), with significant reductions after the treatment (*p* = 0.019 and *p* = 0.009, respectively). No significant differences were found in either the sensation of dry mouth scores (VAS) (*p* = 0.054) or overall saliva production (*p* = 0.477). Lastly, no changes were observed in the Epworth scale (*p* > 0.4).(Table 1).

The mixed-effects models showed that the only significant fixed effect corresponded to the factor time on the pain/burning VAS variable (F = 19.42; *p* < 0.001), indicating a clear reduction in pain after the treatment. Neither the number of locations (>1 vs. 1) nor the development time was significantly associated with the results, which suggests that these factors did not have an influence on the response to the treatment. No significant effects were detected of time with dry mouth VAS (*p* = 0.224) or overall saliva production (*p* = 0.693). (Table 2).

In the “have you noticed an improvement” question in the subjective assessment, the mean was 6 (IQR 2–7) and the improvement was perceived with a mean of the 5th day of treatment (IQR 2–7), with most of the patients experiencing relief within the first week. The treatment was well tolerated. In total, 63.2% of the patients did not report any adverse effects. The most frequent mild events were light sleepiness (10.5%) and mild dysgeusia.

## 4. Discussion

This retrospective study suggests that 1% topical amitriptyline may provide clinical benefit and could represent an effective and well-tolerated therapeutic option for patients with burning mouth syndrome (BMS) in routine clinical practice. After four weeks of treatment, patients showed a significant reduction in oral pain or burning, together with improvements in anxiety and depression scores. However, these psychological findings should be interpreted with caution, as they may reflect the reduction in oral symptoms and overall clinical improvement rather than a direct effect of treatment on psychological status [23].

Although dry mouth scores initially decreased on day 15, this effect was less evident on day 30. This pattern may be related to a transient symptomatic improvement, interindividual variability, or fluctuations in the subjective perception of xerostomia over time [21]. Consistently, no significant changes were observed in objective salivary flow measurements. Overall, these findings support the potential role of topical amitriptyline as a symptomatic treatment option for BMS, particularly for pain relief, while further prospective controlled studies are needed to confirm its long-term efficacy and safety.

The systemic use of amitriptyline has been shown to be efficient in many studies on BMS [8,9,10,11,12]. Table 3 shows a summary of the main clinical studies published on the use of amitriptyline in BMS, both orally and topically, to better contextualize the findings from the present work. Fenelon et al. (2017), Kawasaki et al. (2018), Suga et al. (2019), Watanabe et al. (2022), and Gonçalves et al. (2024) [8,9,10,11,12].described treatment response rates that oscillated between 53% and 76%. However, in most of the cases, significant adverse effects were observed, such as sleepiness, xerostomia, and constipation, which conditioned therapeutic adherence, especially among older adults or those under polypharmacy. These secondary effects are still some of the main barriers against their sustained use for BMS.

In contrast, the topical amitriptyline formulation surges as an attractive alternative due to its comparable tolerability and efficacy [7,14,15,16,17,18]. In our study, the significant reduction in pain was accompanied by a fast initial action and a low incidence of mild adverse effects (sleepiness and dysgeusia), without severe events reported. These findings coincide with the results from Hussein and El-Marssafy [16], who, in a randomized clinical trial, showed that the topical application of different concentrations significantly reduced pain and improved quality of life without provoking important adverse effects. Similarly, Lebel et al. [14,15] observed a mean reduction in pain close to 40%, and a clinically significant improvement (≥50%) in approximately half of the treated patients, confirming a favorable safety profile and a sustained efficacy.

From a physiopathological perspective, the action of topical amitriptyline could be related to the local modulation of sodium and potassium channels, as well as the TRPV1 and NMDA receptors, involved in the peripheral neuronal hyperexcitability that is characteristic of BMS. Nagamine [17,18] has suggested that the drug also acts on central serotonergic, noradrenergic, and dopaminergic pathways, leading to a re-equilibration in the neurotransmission of pain without the need for high systemic concentrations. In our study, the absence of changes in salivary flow and daytime sleepiness supports the hypothesis of a predominantly local effect. The observed improvement in the emotional parameters could be explained, in part, by the interaction between the reduction in pain and psychological well-being derived from the alleviation of the symptoms, instead of the direct effect of the drug on mood.

In addition, it cannot be excluded that part of the observed clinical and emotional improvement was influenced by non-pharmacological factors, particularly a possible placebo effect related to treatment expectations [24,25,26]. This may be especially relevant in burning mouth syndrome, where symptoms are subjective and may fluctuate over time. Previous studies have reported placebo responses in patients with BMS, suggesting that part of the observed improvement may be related to nonspecific factors such as clinical attention or the natural course of symptoms. Therefore, these findings should be interpreted with caution.

### Limitations and Considerations

Despite the favorable results, the limitations inherent to the retrospective design, the moderate sample size, and the absence of a control group and the exploratory nature of this study must be considered. These factors restrict the possibility of establishing causality. Controlled and multi-center studies are needed, with a prolonged follow-up, which will allow us to confirm the efficacy of topical amitriptyline, to define standardized protocols, and to determine their role within the multimodal approach to BMS.

## 5. Conclusions

These preliminary findings suggest that 1% topical amitriptyline may be a useful and well-tolerated option for reducing pain in patients with burning mouth syndrome (BMS), particularly in those who cannot take oral medications or who are under polypharmacy. Although further controlled studies are required to confirm its efficacy and long-term benefits, the present results indicate that the topical route could play a meaningful role in the comprehensive management of BMS.

## Figures and Tables

**Table 1:** Description and comparison of the scale scores according to visit.

Variable	Baseline Median (IQR)	Day 15 Median (IQR) *	Day 30 Median (IQR)	*p*-Value
VAS pain/burning	7.5 (6–9)	5 (5–7)	6 (5–7)	<0.001
VAS dry mouth	6 (3–8)	2.5 (1–4)	5 (2–7)	0.054
HADS-Depression	4 (4–6)	—	1 (1–5)	0.009
HADS-Anxiety	7 (5–11)	—	4 (2–9)	0.019
Epworth Sleepiness Scale	3 (0–7)	—	3 (2–8)	0.950
Unstimulated whole saliva (mm)	30.5 (20–35)	—	28.5 (25–34)	0.477
VAS unpleasant taste	—	0 (0–3)	0.5 (0–6)	0.413
VAS ease of use	—	10 (10–10)	10 (10–10)	0.593
VAS taste	—	0 (0–1)	0 (0–1)	0.458

* (Interquartile range: IQR).

**Table 2:** Mixed-effect models analysis.

Variable	VAS Pain/Burning F	*p*-Value	VAS Dry Mouth F	*p*-Value	Overall Saliva F	*p*-Value
Time	19.42	<0.001	1.56	0.224	0.16	0.693
Number of locations (>1 vs. 1)	0.01	0.908	0.96	0.336	2.28	0.145
Symptom duration (years)	0.21	0.652	0.06	0.808	0.28	0.601

Abbreviations: VAS, visual analog scale. Values shown correspond to F statistics and *p*-values.

**Table 3:** Key studies on amitriptyline in burning mouth syndrome (BMS).

Study	Country	Design	N	Intervention	Duration	Dose/Concentration	Main Findings	Adverse Effects	Evidence Level
**Systemic amitriptyline (oral route)**									
Fenelon et al., 2017 [8]	France	Comparative retrospective	40	Clonazepam 1 mg/day vs. amitriptyline	3 months	10 mg/day	Significant pain reduction; slower onset than clonazepam	Asthenia, dry mouth, somnolence	Observational
Kawasaki et al., 2018 [9]	Japan	Retrospective	27	Oral amitriptyline	1 month	22.1 +/− 10.2 mg/day	70.4% responders; increased salivary flow in non-responders	Xerostomia, somnolence	Observational
Suga et al., 2019 [10]	Japan	Retrospective cohort	187	Oral amitriptyline	≥1 month	13–20 mg/day according to age	76% improvement (PGIC ≥ 3)	Somnolence, dry mouth, constipation	Observational
Watanabe et al., 2022 [11]	Japan	Comparative retrospective	40 (≥80 years)	Amitriptyline vs. aripiprazole	Variable	10 mg/day	Better efficacy (53.8%) and adherence with amitriptyline	Constipation, dizziness, dry mouth	Observational
Goncalves et al., 2024 [12]	Brazil	Case series	35	Oral amitriptyline	Variable	25–50 mg/day	74.2% responders; better response among men	Xerostomia, somnolence	Case series
**Topical amitriptyline**									
Lebel et al., 2026 [14]	Canada	Retrospective real-world practice	15	Topical amitriptyline gel	8 weeks	1% compounded formulation	Mean pain reduction of −3.1/10; 50% achieved ≥ 50% relief	Mild somnolence, dysgeusia, dryness	Observational
Lebel et al., 2024 [15]	France	Retrospective	15	Topical amitriptyline	8 weeks	Not specified	Significant pain reduction and good tolerance	Mild xerostomia, somnolence	Observational
Hussein & El-Marssafy, 2025 [16]	Egypt/Saudi Arabia	Randomized clinical trial	60	Amitriptyline rinse vs. placebo	8 weeks	10 or 25 mg/100 mL	Improvement in NPRS and OHIP-14; dose-dependent effect	No adverse effects reported	RCT
Nagamine, 2024–2025 [17,18]	Japan	Case reports	-	Low-dose topical amitriptyline	Variable	Not detailed	Clinical improvement in isolated cases	Not reported	Case reports

Abbreviations: BMS, burning mouth syndrome; PGIC, Patient Global Impression of Change; NPRS, Numeric Pain Rating Scale; OHIP-14, Oral Health Impact Profile-14; RCT, randomized controlled trial; n.r., not reported.

## Data Availability

The original contributions presented in this study are included in the article. Further inquiries can be directed to the corresponding authors.
